# Detecting chronic kidney disease in population-based administrative databases using an algorithm of hospital encounter and physician claim codes

**DOI:** 10.1186/1471-2369-14-81

**Published:** 2013-04-05

**Authors:** Jamie L Fleet, Stephanie N Dixon, Salimah Z Shariff, Robert R Quinn, Danielle M Nash, Ziv Harel, Amit X Garg

**Affiliations:** 1 Department of Medicine, Division of Nephrology, Western University, London, Canada; 2 Department of Epidemiology & Biostatistics, Western University, London, Canada; 3 Institute for Clinical Evaluative Sciences, Ontario, Canada; 4 Departments of Medicine & Community Health Sciences, University of Calgary, Calgary, Canada; 5 Division of Nephrology, University of Toronto, Toronto, Canada; 6 London Kidney Clinical Research Unit, Room ELL-101, Westminster, London Health Sciences Centre, 800 Commissioners Road East, London, Ontario, N6A 4G5, Canada

**Keywords:** International Classification of Diseases, Validation, Sensitivity, Specificity, Chronic kidney disease, Serum creatinine, eGFR, Validity

## Abstract

**Background:**

Large, population-based administrative healthcare databases can be used to identify patients with chronic kidney disease (CKD) when serum creatinine laboratory results are unavailable. We examined the validity of algorithms that used combined hospital encounter and physician claims database codes for the detection of CKD in Ontario, Canada.

**Methods:**

We accrued 123,499 patients over the age of 65 from 2007 to 2010. All patients had a baseline serum creatinine value to estimate glomerular filtration rate (eGFR). We developed an algorithm of physician claims and hospital encounter codes to search administrative databases for the presence of CKD. We determined the sensitivity, specificity, positive and negative predictive values of this algorithm to detect our primary threshold of CKD, an eGFR <45 mL/min per 1.73 m^2^ (15.4% of patients). We also assessed serum creatinine and eGFR values in patients with and without CKD codes (algorithm positive and negative, respectively).

**Results:**

Our algorithm required evidence of at least one of eleven CKD codes and 7.7% of patients were algorithm positive. The sensitivity was 32.7% [95% confidence interval: (95% CI): 32.0 to 33.3%]. Sensitivity was lower in women compared to men (25.7 vs. 43.7%; p <0.001) and in the oldest age category (over 80 vs. 66 to 80; 28.4 vs. 37.6 %; p < 0.001). All specificities were over 94%. The positive and negative predictive values were 65.4% (95% CI: 64.4 to 66.3%) and 88.8% (95% CI: 88.6 to 89.0%), respectively. In algorithm positive patients, the median [interquartile range (IQR)] baseline serum creatinine value was 135 μmol/L (106 to 179 μmol/L) compared to 82 μmol/L (69 to 98 μmol/L) for algorithm negative patients. Corresponding eGFR values were 38 mL/min per 1.73 m^2^ (26 to 51 mL/min per 1.73 m^2^) vs. 69 mL/min per 1.73 m^2^ (56 to 82 mL/min per 1.73 m^2^), respectively.

**Conclusions:**

Patients with CKD as identified by our database algorithm had distinctly higher baseline serum creatinine values and lower eGFR values than those without such codes. However, because of limited sensitivity, the prevalence of CKD was underestimated.

## Background

Chronic kidney disease (CKD) is a permanent reduction in kidney function that can progress to end stage renal disease (ESRD) requiring either ongoing dialysis or a kidney transplant to maintain life. CKD also affects how many medications are eliminated from the body [1]. In routine practice a laboratory serum creatinine value is used to estimate kidney function by incorporating it into a formula to estimate the glomerular filtration rate and establish whether a patient has CKD. However, in some situations there is an interest in identifying CKD using healthcare database codes when laboratory values are not available. Codes in these databases have their limitations and knowledge of their validity guides their optimal use [2]. The purpose of the current study was to develop and examine the validity of algorithms using hospital encounter (International Classification of Diseases, 10^th^ revision [ICD-10]) codes and physician claim codes for the detection of CKD assessed against a reference standard of estimated glomerular filtration rate (eGFR) determined with laboratory values. We hypothesized, based on validations done for other laboratory-based conditions by our research group, that our best performing algorithm would demonstrate limited sensitivity but excellent specificity for the identification of CKD [3-6]. As suggested in the literature, we also hypothesized that code sensitivity would be lower in women compared to men, and lowest in the oldest age category [7].

## Methods

### Study design

We conducted a retrospective, population-based validation study using linked administrative databases housed at the Institute for Clinical Evaluative Sciences (ICES). The province of Ontario, Canada has approximately 13 million residents, 14% of whom are 65 years of age or older [8]. Residents of Ontario have universal access to hospital care and physician services and those 65 years of age or older have universal prescription drug coverage. Due to the availability of laboratory data, this study was restricted to those individuals aged 65 and older in Southwestern Ontario, which consisted of 80,000 individuals in the year 2006 [9]. We gathered serum creatinine values from 12 regional hospitals in Southwestern Ontario as well as outpatient laboratories. We examined the validity of our primary database algorithm consisting of ICD-10 and physician claims diagnostic codes for detecting CKD by comparing it to eGFR (derived from serum creatinine laboratory values) as the reference standard. Codes of our primary database algorithm were finalized after testing several codes alone or in combination (described below). The reporting of this study follows guidelines for studies of diagnostic accuracy (Additional file 1) [10]. We conducted our study according to a pre-specified protocol that was approved by the institutional review board at Sunnybrook Health Sciences Centre (Toronto, Ontario).

### Data sources

We ascertained the presence of relevant comorbidities for exclusions and baseline characteristics using patient records from seven linked databases. The Ontario Drug Benefit (ODB) Plan database contains records of prescriptions for individuals 65 years or older from outpatient pharmacies. The dispensing of medications for patients aged 65 and older is accurately recorded in this database with an error rate of <1% [11]. The Canadian Institute for Health Information (CIHI) National Ambulatory Care Reporting System (NACRS) contains ambulatory care information on emergency room visits, outpatient procedures, and day surgeries. The CIHI Discharge Abstract Database (CIHI-DAD) captures procedures and diagnoses for hospitalized patients in Ontario. The Ontario Health Insurance Plan (OHIP) database contains all physician claims for medical services covered under the provincial health insurance plan. Lastly, the Registered Persons Database (RPDB) contains demographic information, such as birth date and sex, for all Ontario residents who have ever been covered by the provincial healthcare plan.

In addition to the administrative databases described above, we also used two laboratory datasets for baseline serum creatinine values. An electronic medical record, Cerner® (Kansas City, Missouri, USA), contains inpatient, outpatient, and emergency department laboratory values for 12 hospitals in Southwestern Ontario [12]. Additionally, Gamma Dynacare provided all outpatient serum creatinine values for patients serviced by this laboratory provider in Southwestern Ontario. We have successfully used these laboratory datasets linked to administrative data in previous studies [13-15].

### Patients

We accrued patients at the time of an outpatient prescription for any medication between July 1, 2007 and December 31, 2010. We focused on outpatient prescriptions based on the assumption that the outpatient setting is most likely to capture individuals at time of clinical stability. In addition, administrative data are often used for pharmacoepidemiologic studies, in which having a prescription is paramount. In Ontario, prescription drug coverage is a universal benefit for patients 65 years of age or older. Almost all older residents in Ontario were expected to have at least one prescription medication dispensed through the provincial drug plan over the accrual period. Patients with multiple prescriptions during the accrual period could only be selected once for cohort entry. To be included, patients must have had at least one serum creatinine laboratory test in the year prior to the prescription date from a laboratory in Southwestern Ontario. Serum creatinine testing amongst older adults is fairly ubiquitous in Ontario, with most individuals having at least one outpatient serum creatinine test in a given year [16]. We considered only patients 66 years of age or older in this study to allow for a minimum of one year of baseline prescription information from the provincial drug plan. We excluded laboratory tests where patients were hospitalized up to two days prior to their prescription date to ensure we captured prescriptions first initiated in the outpatient setting. As we were interested in detecting CKD prior to any treatment for kidney failure, we also excluded patients who received dialysis in the year prior or a kidney transplant in the five years prior to the prescription date.

### CKD Database algorithm (Diagnostic test)

Diagnostic codes and their associated attributes are recorded in health administrative databases. For hospital encounters in Canada, trained coders follow specific rules and guidelines set out when assigning diagnostic codes based on a patient’s chart. They cannot interpret any diagnostic tests, such as x-rays or laboratory values, unless a diagnosis is specifically written by the physician in the medical chart [17]. For physician claims, typically clerical personnel employed by a physician submit billing and diagnosis codes for each patient seen in order for the physician to be paid for services provided.

To develop our algorithm to detect CKD two nephrologists reviewed all available ICD-10 and OHIP diagnostic database codes and identified 55 that were deemed potentially relevant (Additional file 1). An individual was classified as algorithm positive for CKD if a code appeared at least once in the five years prior to the outpatient prescription date. We finalized elements of our primary database algorithm using a process described below (see ‘Data Analysis’ section).

### Kidney function laboratory values (Reference standard)

GFR was estimated from serum creatinine using the CKD-EPI equation [18,19]. We chose the most recent serum creatinine value from any setting prior to the outpatient prescription as our test value. If this value was done while a patient was in hospital, this was the most recent value prior to discharge (which helps avoid temporary elevated serum creatinine values due to acute kidney injury where physicians wait for resolution prior to hospital discharge).

Although a recently revised staging system for CKD does exist its use in the elderly remains controversial [20]. Nonetheless, stage IIIb CKD or more is defined by an estimated glomerular filtration rate (eGFR) <45 mL/min per 1.73 m^2^ and is accepted as a clinically important reduction in kidney function in elderly patients [21]. Furthermore, when creatinine tests are repeated, eGFR values <45 mL/min per 1.73 m^2^ in the outpatient setting are more stable compared to higher eGFR values [22]. For these reasons we selected our primary threshold to define CKD as an eGFR <45 mL/min per 1.73 m^2^. However, in additional analyses we also examined thresholds of <60 and <30 mL/min per 1.73 m^2^. We used thresholds rather than mutually exclusive groups to mimic how inferences about the types of patients detected by the algorithm will be made in practice.

### Data analysis

We calculated the sensitivity, specificity, positive predictive value, and negative predictive value of each coding algorithm for each eGFR threshold. We developed a two by two contingency table for each code to assess its validity for detecting CKD (see Additional file 1 for an example). We examined the validity of each code on its own or combined within an algorithm using the Boolean operator “OR”. For our final primary algorithm we combined codes to include those that either had a sensitivity of at least 3% or were conceptually similar to included codes. Because serum creatinine and eGFR are continuous variables, we also contrasted the mean, median, and interquartile ranges of serum creatinine and eGFR values for those who were CKD algorithm positive (i.e. presence of at least one of codes in our final algorithm) compared to those who were CKD algorithm negative (i.e. absence of all of the codes in our final algorithm). We performed additional analyses to further understand the attributes of the CKD algorithm. These included restricting to outpatient only laboratory values, assessing at least two codes in the look-back window, and applying different combinations of renal codes. We calculated 95% confidence intervals (CI) for single proportions using the Wilson Score method [22]. We expressed serum creatinine and eGFR values as medians with interquartile ranges (IQR). We performed all analyses using SAS version 9.2 (SAS Institute Incorporated, Cary, North Carolina, USA, 2008).

## Results

Our study included 123,499 patients (patient selection presented in Additional file 1). Patient baseline characteristics are presented in Table 1. The median age was 75 years and 54% of the patients were women. Approximately 23% of our cohort had diabetes, while 40% had coronary artery disease. A total of 15.4% of patients had a measured eGFR study value of <45 mL/min per 1.73 m^2^. Seventy five percent of serum creatinine values used to estimate GFR were taken from an outpatient laboratory, with the remaining coming from a hospital setting. Of note, 52,081 (42.2%) patients also had an additional serum creatinine test in the 90 to 365 days prior to their study serum creatinine test (average 255 days). The absolute median (IQR) difference in eGFR between the study and prior test was minimal confirming stability of eGFR [5 mL/min per 1.73 m^2^ (2 to 11 mL/min per 1.73 m^2^)]. Our final CKD algorithm consisted of 11 codes (with the performance of individual codes and other algorithms presented in Table 2 and Additional file 1). This was an efficient algorithm, as the sensitivity decreased by less than 1.5% while there was an increase in both specificity and positive predictive value compared to the algorithm that used any one of the 55 codes (results of the algorithm of any 55 codes presented in Additional file 1). Using our final algorithm, a total of 9501 (7.7%) patients were classified positive for the CKD database algorithm. The sensitivity of the CKD algorithm for the detection of an eGFR <45 mL/min per 1.73 m^2^ was 32.7% (95% CI: 32.0 to 33.3%) and the specificity was 96.9% (95% CI: 96.7 to 97.0%). The positive predictive value was 65.4% (95% CI: 64.4 to 66.3%) and negative predictive value was 88.8% (95% CI: 88.6 to 89.0%). When the cohort was restricted to patients with an outpatient serum creatinine value only, we found the performance of the algorithm was not appreciably different (see Additional file 1). Additionally, when at least two codes were required in the look-back window, the sensitivity decreased by approximately 14%, however the positive predictive value increased compared to when at least one code was used.

**Table 1:** Baseline characteristics

	**N = 123499**
**Demographics**	
Age, years, *median (IQR)*	75 (70–81)
Women, *n (%)*	67207 (54.4)
**Income quintile, *n (%)***	
One (lowest)	24803 (20.1)
Two	24607 (19.9)
Three (middle)	25016 (20.3)
Four	23112 (18.7)
Five (highest)	24837 (20.1)
**Rural Location, *n (%)***	25707 (20.8)
**Comorbidities, *n (%)***	
Coronary artery disease^¶^	49027 (39.7)
Diabetes mellitus^£^	28759 (23.3)
Heart failure	20259 (16.4)
Peripheral vascular disease	2730 (2.2)
Stroke/Transient ischemic attack	4812 (3.9)
**Medication use in prior 6 months, *n (%)***	
Angiotensin-converting enzyme inhibitors	49635 (40.2)
Angiotensin-receptor blockers	28438 (23.0)
Beta blockers	47051 (38.1)
Calcium channel blockers	40600 (32.9)
Loop diuretics	22860 (18.5)
NSAIDs(excluding aspirin)	26478 (21.4)
Warfarin	14967 (12.1)
Potassium sparing diuretics	9948 (8.1)
Statins	65168 (52.8)
Thiazide diuretics	27448 (22.2)
Baseline Laboratory Measurements*	
Serum creatinine μmol/L, *median (IQR)*	84 (70–102)
eGFR category ^Ŧ^mL/min per 1.73m^2^, *n (%)*	
≥60mL	78584 (63.6)
45-59	25904 (21.0)
30-44	13749 (11.1)
15-29	4624 (3.7)
<15	638 (0.5)
Serum sodium mmol/L, *median, (IQR)*	140 (138–142)
Serum potassium mmol/L, *median (IQR)*	4.2 (3.9-4.6)
Proteinuria (dipstick value) g/L	
Category, *n (%)*	
Negative	23792 (19.3)
0.3	1509 (1.2)
1.0	962 (0.8)
≥3.0	220 (0.2)

Abbreviations: IQR, interquartile range; NSAIDs, non-steroidal anti-inflammatory drugs; eGFR, estimated glomerular filtration rate.

^¶^ Coronary artery disease includes receipt of coronary artery bypass graft surgery, percutaneous coronary intervention and diagnoses of angina.

^£^ Assessed by diabetic medication use in previous 6 months.

* All patients had a serum creatinine measurement. A total of 109,743 (88.9%), and 111,304 (90.1%) patients had a serum sodium and potassium measurement in the year prior to cohort entry, respectively while 26,483(21.4%) patients had a urine dipstick protein value. The most recent values prior to the prescription date were used.

^Ŧ^eGFR was calculated using the CKD-Epi equation.

CKD-Epi equation:141 x min([serum creatinine in umol/L /88 · 4 ]/κ, 1)^α^ x max([serum creatinine in umol/L / 88 · 4]/κ, 1)^-1·209^ x 0 · 993^Age^ x 1 · 018 [if female] x 1 · 159 [if African American] *κ = 0 · 7 for females and 0 · 9 for males, α = −0 · 329 for females and −0 · 411 for males, min = the minimum of Scr/κ or 1, max = the maximum of Scr/κ or 1.Racial information was not available in our data sources and all patients were assumed not to be of non African-Canadian race. This was a reasonable assumption; as of 2006, African-Canadians represented <7% of the Ontario population. Source:http:// http://www12.statcan.ca/census-recensement/2006/dp-pd/hlt/97-562/index.cfm?Lang=E .*

**Table 2:** Performance of each of the 11 codes in the final CKD algorithm

**Code**	**Description**	**Sensitivity**	**Specificity**	**PPV**	**NPV**
ICD10 E102	Type 1 diabetes mellitus with incipient diabetes nephropathy adequately or inadequately controlled by insulin, diet, or oral agents	0.17%	99.99%	69.57%	84.63%
ICD10 E112	Type 2 diabetes mellitus with incipient diabetes nephropathy adequately or inadequately controlled by insulin, diet, or oral agents	5.55%	99.42%	63.73%	85.26%
ICD10 E132	Other specified diabetes mellitus with incipient diabetes nephropathy adequately or inadequately controlled by insulin, diet, or oral agents	0.01%	100.00%	100.00%	84.61%
ICD10 E142	Unspecified diabetes mellitus with incipient diabetes nephropathy adequately or inadequately controlled by insulin, diet, or oral agents	0.86%	99.92%	67.49%	84.71%
ICD10 I12	Hypertensive renal disease	7.20%	99.60%	76.64%	85.50%
ICD10 I13	Hypertensive renal and heart disease	0.49%	99.98%	78.15%	84.67%
ICD10 N08	Glomerular disorders in diseases classified elsewhere	3.75%	99.75%	73.48%	85.07%
ICD10 N18	Chronic renal failure	12.24%	99.45%	80.08%	86.17%
ICD10 N19	Unspecified renal failure	4.51%	99.58%	66.38%	85.14%
OHIP DX 403	Hypertensive renal disease	3.08%	99.74%	68.02%	84.98%
OHIP DX 585	Chronic renal failure, uremia	22.39%	98.20%	69.35%	87.43%

Abbreviations: ICD10, International Classification of Diseases, 10^th^ revision; OHIP DX, Ontario Health Insurance Plan diagnostic codes; PPV, positive predictive value; NPV, negative predictive value. Results are presented for the detection of an eGFR < 45 mL/min per 1.73 m^2^.

The validity of the algorithm for detection of the additional eGFR thresholds (<60 and <30 mL/min per 1.73 m^2^) is presented in Table 3. Algorithm sensitivity increased to 58.8% for the detection of more pronounced CKD (eGFR <30 mL/min per 1.73 m^2^).

**Table 3:** Diagnostic performance characteristics of the final algorithm for other thresholds of chronic kidney disease

	**<60 ml/min per 1.73 m**^**2**^	**<30 ml/min per 1.73 m**^**2**^
	% (95% CI)	% (95% CI)
**Sensitivity**	18.0 (17.7-18.4)	58.8 (57.4-60.1)
**Specificity**	98.2 (98.1-98.3)	94.6 (94.5-94.7)
**PPV**	85.2 (84.5-85.9)	32.5 (31.6-33.5)
**NPV**	67.7 (67.4-68.0)	98.1 (98.0-98.2)

Abbreviations: PPV, positive predictive value; NPV, negative predictive value; CI, confidence interval.

Among patients who were positive for our final CKD coding algorithm the median (IQR) serum creatinine value was 135 μmol/L (106 to 179 μmol/L) and for those who were algorithm negative, the value was 82 μmol/L (69 to 98 μmol/L). Similar trends were seen with eGFR; algorithm positive patients had an eGFR of 38 ml/min per 1.73 m^2^ (26 to 51 ml/min per 1.73 m^2^) and algorithm negative patients had a value of 69 ml/min per 1.73 m^2^ (56 to 82 ml/min per 1.73 m^2^).

Performance of the CKD algorithm stratified by age and sex is presented in Table 4. Algorithm sensitivity was lower in women compared to men (25.7 vs. 43.7%; p <0.001) and in the oldest patients (over 80 vs. 66 to 80; 28.4 vs. 37.6%; p <0.001). Men who were algorithm positive had a median (IQR) eGFR similar to algorithm positive women [40 (28 to 53) vs. 36 (25 to 49) mL/min per 1.73 m^2^]. Patients in the oldest age category (>80 years) who were positive for the CKD algorithm had a median (IQR) eGFR of 34 mL/min per 1.73 m^2^ (25 to 45 mL/min per 1.73 m^2^) compared to 41 ml/min per 1.73 m^2^ (29 to 55 mL/min per 1.73 m^2^) in algorithm positive patients in the second oldest age category (66 to 80 years).

**Table 4:** Performance of the CKD algorithm by age and sex

	**Sensitivity**	**Specificity**	**PPV**	**NPV**
	**% (95% CI)**	**% (95% CI)**	**% (95% CI)**	**% (95% CI)**
**66-80 years old**	37.6 (36.6-38.6)	97.1 (97.0-97.2)	59.1 (57.9-60.4)	93.3 (93.1-93.5)
**>80 years old**	28.4 (27.5-29.2)	96.1 (95.8-96.3)	74.5 (73.1-75.8)	76.8 (76.4-77.3)
	**Code Positive**		**Code Negative**	
	*eGFR (mL/min per 1.73m*^*2*^*)*	*Creatinine (μmol/L)*	*eGFR (mL/min per 1.73m*^*2*^*)*	*Creatinine (μmol/L)*
**66-80 years old**	41 (29–55)	133 (104–176)	73 (60–85)	81 (69–95)
**>80 years old**	34 (25–45)	140 (109–183)	58 (46–72)	87 (71–106)
	**Sensitivity**	**Specificity**	**PPV**	**NPV**
	% (95% CI)	% (95% CI)	% (95% CI)	% (95% CI)
**Men**	43.7 (42.5-44.8)	96.0 (95.8-96.2)	62.0 (60.6-63.3)	91.9 (91.7-92.1)
**Women**	25.7 (24.9-26.5)	97.6 (97.5-97.7)	69.4 (68.0-70.8)	86.2 (86.0-86.5)
	**Code Positive**		**Code Negative**	
	*eGFR (mL/min per 1.73m*^*2*^*)*	*Creatinine (μmol/L)*	*eGFR (mL/min per 1.73m*^*2*^*)*	*Creatinine (μmol/L)*
**Men**	40 (28–53)	145 (115–190)	72 (59–83)	90 (79–106)
**Women**	36 (25–49)	123 (95–164)	67 (53–81)	74 (64–89)

Abbreviations: ICD-10, International Classification of Diseases, 10^th^ revision; OHIP, Ontario Health Insurance Plan; PPV, positive predictive value; NPV, negative predictive value; CI, confidence interval.

Dichotomous results are presented for a reference eGFR < 45 mL/min per 1.73 m^2^.

Numbers listed are median (interquartile range).

Creatinine was measured in serum.

## Discussion

We developed and assessed the accuracy and validity of algorithms that used hospital encounter and physician claim codes from population-based administrative data in Ontario, Canada to detect CKD. Older patients identified as having CKD by the final database algorithm had higher serum creatinine values and lower eGFR values than those without such codes. Similar to previous studies, our final algorithm demonstrated a high specificity and negative predictive value [6,7]. For example, the specificity was high (>92%) for all eGFR thresholds and stratified analyses. The high negative predictive value (88.8% for the main eGFR threshold) provides confidence that individuals who are algorithm negative most likely do not have CKD.

The range of sensitivities reported in other CKD validation studies has been broad [6,7]. Our algorithm demonstrated 33% sensitivity for detecting an eGFR <45 mL/min pre 1.73 m^2^, and resulted in an appreciable underestimation of disease. In both the current study and a recent study done in Alberta, Canada sensitivity was lowest for detecting milder forms of CKD and improved with disease severity [7]. As coding relies on recorded diagnoses, physicians may be less likely to recognize or act on mild CKD. As well, in both studies algorithm sensitivity was lower in women compared to men, and in older compared to younger elderly patients. These are segments of the population where CKD has traditionally been unrecognized [23]. Differences in code validity by age and sex may lead to biased estimates when assessing CKD risk in certain populations using the database algorithm. In general, the algorithm seems most useful when assessing CKD as a baseline characteristic and when it is not a main variable in an analysis. Given limits in sensitivity, the CKD coding algorithm is also less useful as an outcome measure.

We recently published a systematic review of 19 studies on the validity of algorithms of healthcare administrative database codes to detect CKD [6]. Across the studies, patients were accrued from 1984 to 2004 and some studies included CKD defined by the receipt of dialysis. Most of the studies used an ICD-9 version of the codes. The four studies validating ICD-10 codes for CKD used the reference standard of chart review. This differs from the current study where the preferred reference standard of laboratory values was employed.

Our study has other strengths. The validation follows guidelines set out for studies of diagnostic accuracy [10]. As well, all individuals in Canada receive universal health care. This provided us with access to information from a large number of patients which resulted in estimates with good precision.

Our study does have some limitations. We used a pragmatic approach to algorithm development and only combined codes using the Boolean operator “OR”. We also only defined codes as absent or present based on a fixed window of being present at least once in the prior five years. While we did also assess our main algorithm looking for two codes in the look-back, we found that the loss in sensitivity was not worth the small gain in positive predictive value. Additional efforts could consider more refined methods of combining codes, and or different algorithms focused on maximizing a single performance measure such as specificity. Combining codes could also be done with machine learning which takes information from a variety of sources in an automated fashion to compile the most efficient algorithms [24].

We were interested in developing an algorithm to detect a reduced eGFR. Estimated GFR is the most important parameter of CKD. In clinical practice, two measurements separated by at least three months are required to confirm its presence, while in this study we assessed eGFR at a single point in time (although the single value was stable in the subset of patients with another baseline measurement). It might be useful in the future to develop different algorithms for different levels of low eGFR. As well, the algorithm may not be useful for detecting CKD defined by proteinuria in the absence of low eGFR.

We did not split our sample into derivation and validation subsets. Rather, to confirm similar performance our final CKD algorithm should be tested in other regions and be re-examined in our region at a future time. It is possible that similar physician claim codes included in the algorithm will not operate well in other regions, particularly in jurisdictions without universal health care or without a fee-for-service model. The algorithm should also be validated in younger patients where CKD is less prevalent and serum creatinine testing is less common [25-27].

Finally, it must be recognized that any algorithm will fail to capture individuals who have CKD but do not have a laboratory test to identify its presence in routine care.

## Conclusion

We have described the diagnostic properties of an algorithm to detect CKD in the large healthcare administrative databases of Ontario, Canada. This algorithm can be used for health services research and population disease surveillance when serum creatinine results are unavailable. However, the algorithm should be used judiciously given its limited sensitivity.

## Competing interests

The authors declare that they have no competing interests.

## Authors’ contributions

JLF participated in the coordination of the study, study design, provided interpretation of study results, and drafted the manuscript. SND participated in the study design, performed the analysis and provided interpretation of study results. SZS contributed to the study design and interpretation of study results. DMN, RRQ, and ZH contributed to the study design and provided feedback on the manuscript. AXG conceived of the study, participated in its design and interpretation, helped draft the manuscript and provided feedback on the manuscript. All authors read and approved the final manuscript.

## Pre-publication history

The pre-publication history for this paper can be accessed here:

http://www.biomedcentral.com/1471-2369/14/81/prepub

## Supplementary Material

Additional file 1: Table S1 Standards for the reporting of diagnostic accuracy studies checklist. **Table S2**. List of all 55 potential chronic kidney disease codes and performance in detecting an estimated glomerular filtration rate of < 45 mL/min per 1.73 m^2^. All potential codes were reviewed by two nephrologists to identify any potentially relevant renal codes. The final list consisted of 11 of these codes. Click here for file
