# Supplementary material for: Detecting chronic kidney disease in population-based administrative databases using an algorithm of hospital encounter and physician claim codes
Source: BMC Nephrol. 2013 Apr 5;14:81. doi: 10.1186/1471-2369-14-81 (PMC3637099; doi:10.1186/1471-2369-14-81)
Supplement: Additional file 1: Table S1 — Standards for the reporting of diagnostic accuracy studies checklist. Table S2. List of all 55 potential chronic kidney disease codes and performance in detecting an estimated glomerular filtration rate of < 45 mL/min per 1.73 m2. All potential codes were reviewed by two nephrologists to identify any potentially relevant renal codes. The final list consisted of 11 of these codes. [file 1471-2369-14-81-S1.doc]

Appendix A

# STARD checklist for reporting of studies of diagnostic accuracy

(version January 2003)

| **Section and Topic** | **Item**  **#** |  | **On page #** |
| --- | --- | --- | --- |
| TITLE/ABSTRACT/  KEYWORDS | 1 | Identify the article as a study of diagnostic accuracy (recommend MeSH heading 'sensitivity and specificity'). | Abstract |
| INTRODUCTION | 2 | State the research questions or study aims, such as estimating diagnostic accuracy or comparing accuracy between tests or across participant groups. | Introduction |
| METHODS |  |  |  |
| *Participants* | 3 | The study population: The inclusion and exclusion criteria, setting and locations where data were collected. | Methods – patients |
|  | 4 | Participant recruitment: Was recruitment based on presenting symptoms, results from previous tests, or the fact that the participants had received the index tests or the reference standard? | Methods –patients |
|  | 5 | Participant sampling: Was the study population a consecutive series of participants defined by the selection criteria in item 3 and 4? If not, specify how participants were further selected. | Methods – patients |
|  | 6 | Data collection: Was data collection planned before the index test and reference standard were performed (prospective study) or after (retrospective study)? | Methods – study design |
| *Test methods* | 7 | The reference standard and its rationale. | Methods – study design |
|  | 8 | Technical specifications of material and methods involved including how and when measurements were taken, and/or cite references for index tests and reference standard. | Methods – study design |
|  | 9 | Definition of and rationale for the units, cut-offs and/or categories of the results of the index tests and the reference standard. | Methods – kidney function laboratory value |
|  | 10 | The number, training and expertise of the persons executing and reading the index tests and the reference standard. | Methods – CKD database algorithm |
|  | 11 | Whether or not the readers of the index tests and reference standard were blind (masked) to the results of the other test and describe any other clinical information available to the readers. | n/a |
| *Statistical methods* | 12 | Methods for calculating or comparing measures of diagnostic accuracy, and the statistical methods used to quantify uncertainty (e.g. 95% confidence intervals). | Methods – data analysis |
|  | 13 | Methods for calculating test reproducibility, if done. | n/a |
| RESULTS |  |  |  |
| *Participants* | 14 | When study was performed, including beginning and end dates of recruitment. | Methods - patients |
|  | 15 | Clinical and demographic characteristics of the study population (at least information on age, gender, spectrum of presenting symptoms). | Results; Table 1 |
|  | 16 | The number of participants satisfying the criteria for inclusion who did or did not undergo the index tests and/or the reference standard; describe why participants failed to undergo either test (a flow diagram is strongly recommended). | Results; Table 1, Appendix D |
| *Test results* | 17 | Time-interval between the index tests and the reference standard, and any treatment administered in between. | Methods - patients; Table 1 footnote |
|  | 18 | Distribution of severity of disease (define criteria) in those with the target condition; other diagnoses in participants without the target condition. | Results; Table 3; Appendix E |
|  | 19 | A cross tabulation of the results of the index tests (including indeterminate and missing results) by the results of the reference standard; for continuous results, the distribution of the test results by the results of the reference standard. | Results; Table 2 |
|  | 20 | Any adverse events from performing the index tests or the reference standard. | n/a |
| *Estimates* | 21 | Estimates of diagnostic accuracy and measures of statistical uncertainty (e.g. 95% confidence intervals). | Results; Tables 3, 4; Appendix E |
|  | 22 | How indeterminate results, missing data and outliers of the index tests were handled. | n/a |
|  | 23 | Estimates of variability of diagnostic accuracy between subgroups of participants, readers or centers, if done. | n/a |
|  | 24 | Estimates of test reproducibility, if done. | n/a |
| DISCUSSION | 25 | Discuss the clinical applicability of the study findings. | Discussion |

Appendix B

| **A list of 55 chronic kidney disease codes and their performance in detecting an estimated glomerular filtration rate < 45 mL/min per 1.73 m^2^** | | | | | |
| --- | --- | --- | --- | --- | --- |
| **Code** | **Description** | **Sensitivity** | **Specificity** | **PPV** | **NPV** |
| ICD10 C64 | Malignant neoplasm of kidney, except renal pelvis | 0.88% | 99.78% | 41.96% | 84.69% |
| ICD10 D300 | Benign neoplasm of kidney | 0.06% | 99.98% | 32.35% | 84.61% |
| ICD10 E102 | Type 1 diabetes mellitus with incipient diabetes nephropathy adequately or inadequately controlled by insulin, diet, or oral agents | 0.17% | 99.99% | 69.57% | 84.63% |
| ICD10 E112 | Type 2 diabetes mellitus with incipient diabetes nephropathy adequately or inadequately controlled by insulin, diet, or oral agents | 5.55% | 99.42% | 63.73% | 85.26% |
| ICD10 E132 | Other specified diabetes mellitus with incipient diabetes nephropathy adequately or inadequately controlled by insulin, diet, or oral agents | 0.01% | 100.00% | 100.00% | 84.61% |
| ICD10 E142 | Unspecified diabetes mellitus with incipient diabetes nephropathy adequately or inadequately controlled by insulin, diet, or oral agents | 0.86% | 99.92% | 67.49% | 84.71% |
| ICD10 M103 | Gout due to impaired renal function | 0.02% | 100.00% | 50.00% | 84.61% |
| ICD10 I12 | Hypertensive renal disease | 7.20% | 99.60% | 76.64% | 85.50% |
| ICD10 I13 | Hypertensive renal and heart disease | 0.49% | 99.98% | 78.15% | 84.67% |
| ICD10 I701 | Atherosclerosis of renal artery | 0.17% | 99.98% | 62.26% | 84.63% |
| ICD10 K767 | Hepatorenal syndrome | 0.01% | 100.00% | 66.67% | 84.61% |
| ICD10 M300 | Polyarteritis syndrome | 0.03% | 100.00% | 62.50% | 84.61% |
| ICD10 M301 | Allergic granulomatous angiitis | 0.00% | 100.00% | N/A | 84.61% |
| ICD10 M308 | Other conditions related to polyarteritis nodosa | 0.00% | 100.00% | N/A | 84.61% |
| ICD10 M310 | Hypersensitivity angiitis | 0.03% | 100.00% | 83.33% | 84.61% |
| ICD10 M311 | Thrombotic microangiopathy | 0.01% | 99.99% | 20.00% | 84.61% |
| ICD 10 M313 | Wegener's granulomatosis | 0.09% | 99.99% | 65.38% | 84.62% |
| ICD10 M32 | Systemic lupus erythematosus | 0.07% | 99.96% | 26.42% | 84.61% |
| ICD10 M34 | Systemic sclerosis | 0.06% | 99.94% | 16.22% | 84.61% |
| ICD10 N00 | Acute nephritic syndrome | 0.05% | 100.00% | 83.33% | 84.61% |
| ICD10 N01 | Rapidly progressive nephritic syndrome | 0.02% | 100.00% | 75.00% | 84.61% |
| ICD10 N03 | Chronic nephritic syndrome | 0.06% | 100.00% | 80.00% | 84.61% |
| ICD10 N04 | Nephritic syndrome | 0.08% | 99.99% | 57.69% | 84.62% |
| ICD10 N05 | Unspecified nephritic syndrome | 0.08% | 100.00% | 83.33% | 84.62% |
| ICD10 N07 | Hereditary nephropathy, not elsewhere classified | 0.00% | 100.00% | N/A | 84.61% |
| ICD10 N08 | Glomerular disorders in diseases classified elsewhere | 3.75% | 99.75% | 73.48% | 85.07% |
| ICD10 N11 | Chronic tubulo-interstitial nephritis | 0.05% | 99.98% | 38.46% | 84.61% |
| ICD10 N14 | Drug- and heavy metal-induced tubulo-interstitial and tubular conditions | 0.12% | 99.99% | 74.19% | 84.62% |
| ICD10 N15 | Other renal tubulo-interstitial diseases | 0.02% | 99.99% | 30.77% | 84.61% |
| ICD10 N16 | Renal tubulo-interstitial disorders in diseases classified elsewhere | 0.03% | 100.00% | 60.00% | 84.61% |
| ICD10 N18 | Chronic renal failure | 12.24% | 99.45% | 80.08% | 86.17% |
| ICD10 N19 | Unspecifed renal failure | 4.51% | 99.58% | 66.38% | 85.14% |
| ICD10 N25 | Disorders resulting from impaired renal tubular function | 0.09% | 100.00% | 77.27% | 84.62% |
| ICD10 N26 | Unspecified contracted kidney | 0.07% | 99.99% | 50.00% | 84.61% |
| ICD10 N27 | Small kidney of unknown cause | 0.00% | 100.00% | 0.00% | 84.61% |
| ICD10 N29 | Other disorders of kidney and ureter in diseases classified elsewhere | 0.00% | 100.00% | 0.00% | 84.61% |
| ICD10 Q611 | Polycystic kidney, autosomal recessive | 0.00% | 100.00% | N/A | 84.61% |
| ICD10 Q612 | Polycystic kidney, autosomal dominant | 0.01% | 100.00% | 100.00% | 84.61% |
| ICD10 Q613 | Polycystic kidney, unspecified | 0.00% | 100.00% | 0.00% | 84.61% |
| ICD10 R80 | Isolated proteinuria | 0.04% | 99.99% | 38.10% | 84.61% |
| ICD10 R944 | Abnormal results of kidney function studies | 0.04% | 100.00% | 72.73% | 84.61% |
| ICD10 N137 | Vesicoureteral-reflux-associated uropathy | 0.01% | 100.00% | 50.00% | 84.61% |
| ICD10 N280 | Ischaemia and infarction of kidney | 0.08% | 99.99% | 55.17% | 84.62% |
| ICD10 N2888 | Other specified disorders of kidney and ureter | 0.18% | 99.95% | 40.00% | 84.62% |
| ICD10 N06 | Isolated proteinuria with diffuse mesangial proliferative glomerulonephritis | 0.00% | 100.00% | N/A | 84.61% |
| ICD10 N391 | Persistent proteinuria, unspecified | 0.02% | 10.00% | 50.00% | 84.61% |
| ICD10 I150 | Renovascular hypertension benign or malignant | 0.02% | 100.00% | 75.00% | 84.61% |
| ICD10 I151 | Hypertension secondary to other renal disorders | 0.01% | 100.00% | 100.00% | 84.61% |
| ICD10 N392 | Orthostatic proteinuria, unspecified | 0.00% | 100.00% | N/A | 84.61% |
| ICD10 N23 | Unspecified renal colic | 0.36% | 99.67% | 16.71% | 84.61% |
| ICD10 N2880 | Hypertrophy of kidney | 0.00% | 100.00% | 0.00% | 84.61% |
| OHIP DX 403 | Hypertensive renal disease | 3.08% | 99.74% | 68.02% | 84.98% |
| OHIP DX 580 | Acute glomerulonephritis | 0.82% | 99.88% | 55.56% | 84.70% |
| OHIP DX 581 | Nephrotic syndrome | 0.54% | 99.91% | 51.78% | 84.66% |
| OHIP DX 585 | Chronic renal failure, uremia | 22.39% | 98.20% | 69.35% | 87.43% |

Appendix C

|  |  | Reference Standard: **Chronic kidney disease** defined by an estimated glomerular filtration rate **<45 mL/min per 1.73m^2^** | |
| --- | --- | --- | --- |
|  |  | **<45 mL/min per 1.73m^2^** | **≥45 mL/min per 1.73m^2^** |
| **Chronic kidney disease algorithm** defined by **ICD-10 and OHIP diagnostic codes** | Code Positive | a | b |
|  | Code Negative | c | d |
| Sensitivity=a/(a+c): the proportion of patients with an estimated glomerular filtration rate **<45 mL/min per 1.73m^2^** who are positive for the CKD algorithm  Specificity=d/(b+d): the proportion of patients with an estimated glomerular filtration rate **≥45 mL/min per 1.73m^2^** who are negative for CKD algorithm  Positive predictive value=a/(a+b): proportion of patients positive for the CKD algorithm who had an estimated glomerular filtration rate **<45 mL/min per 1.73m^2^**  Negative predictive value=d/(c+d): proportion of patients negative for the CKD algorithm who had an estimated glomerular filtration rate **≥45 mL/min per 1.73m^2^** | | | |

Appendix D

Patients with evidence of an outpatient prescription and at least one serum creatinine laboratory test* between July 1^st^ 2007 and December 31^st^ 2010

N = 161,988

Patients excluded from study

Age <66 on prescription date: 36,978

Death prior to prescription date: 53

Evidence of dialysis in 1 year prior to prescription date: 820

Evidence of renal transplant in 5 years prior to prescription date: 166

Evidence of hospital discharge in 2 days prior to prescription date: 472

Patients included in the study

N = 123,499

* Serum creatinine laboratory test must have been within one year prior to prescription date. Serum creatinine values <10 µmol/L and >2500 µmol/L were considered data errors and were excluded

Appendix E

| **Performance of other algorithms to detect an eGFR < 45 mL/min per 1.73 m^2^** | | | | |
| --- | --- | --- | --- | --- |
|  | **Sensitivity**  % (95% CI) | **Specificity**  % (95% CI) | **PPV**  % (95% CI) | **NPV**  % (95% CI) |
| **N18 only** | 12.2 (11.8-12.7) | 99.5 (99.4-99.5) | 80.1 (78.6-81.5) | 86.2 (86.0-86.4) |
| **ICD10 codes only** | 17.6 (17.1-18.2) | 98.5 (98.4-98.5) | 67.4 (66.1-68.7) | 86.8 (86.6-87.0) |
| **OHIP codes only** | 24.1 (23.5-24.7) | 97.8 (97.3-97.9) | 66.8 (65.7-67.9) | 87.6 (87.4-87.8) |
| **All codes** | 34.0 (33.3-34.7) | 96.0 (95.9-96.1) | 60.6 (59.7-61.5) | 88.9 (88.7-89.1) |
| Abbreviations: eGFR estimated glomerular filtration rate; ICD-10, International Classification of Diseases, 10^th^ revision; OHIP, Ontario Health Insurance Plan; PPV, positive predictive value; NPV, negative predictive value; CI, confidence interval.  Numbers listed are median (interquartile range).  Creatinine was measured in serum.  ICD10 codes only consisted of the following codes: I12, I13, N01, N03, N05, N07, N14, N15, N00, N04, N08, N18, N91, N26, N25, N137, N280, N2888, N06, N391  OHIP codes only consisted of the following codes: 403, 580, 581, 585  All codes consisted of the 55 codes listed in Appendix B | | | | |

Appendix F

| **Performance of the primary algorithm to detect an eGFR < 45 mL/min per 1.73 m^2^ when the cohort was restricted to patients with an outpatient baseline serum creatinine value** (cohort size 92,861 individuals) | | | | |
| --- | --- | --- | --- | --- |
|  | **Sensitivity**  % (95% CI) | **Specificity**  % (95% CI) | **PPV**  % (95% CI) | **NPV**  % (95% CI) |
| **Primary algorithm** | 34.9 (34,1-35.7) | 97.1 (96.9-97.2) | 66.9 (65.8-68.0) | 89.7 (89.5-89.9) |
